# Renal Involvement in Primary Sjögren's Syndrome: A Multi-centric Study From North India

**DOI:** 10.7759/cureus.86494

**Published:** 2025-06-21

**Authors:** Scienthia Sanjeevani, Pallavi Prasad, Sourabh Sharma, Sahil Bagai, Dinesh Khullar

**Affiliations:** 1 Nephrology, Kalinga Institute of Medical Sciences, Bhubaneswar, IND; 2 Nephrology, Vardhman Mahavir Medical College (VMMC) and Safdarjung Hospital, New Delhi, IND; 3 Nephrology, Max Super Speciality Hospital Saket New Delhi, New Delhi, IND

**Keywords:** chronic tubulointerstitial disease, connective tissue disorder, kidney biopsy, renal tubular acidosis (rta), sjogren's disease

## Abstract

Introduction

Renal involvement in primary Sjögren's syndrome is often underdiagnosed and overlooked. There is a lack of substantial data on the renal manifestations and outcomes in these patients. This study aims to stress the importance of thorough evaluation of renal dysfunction in individuals with Sjögren's syndrome to improve their overall health and quality of life.

Methods

This multicentric, retrospective study analyzed patients with renal involvement in Primary Sjögren's syndrome from 2013 to 2021. Clinical profiles of those affected by primary Sjögren's syndrome and renal involvement were examined. Patients were enrolled based on specific inclusion criteria, and relevant biochemical tests and kidney biopsy results were reviewed. One-year follow-up data was also included.

Results

A total of 38 patients were studied, with a strong female predominance (N=36, 94.7%) and a mean age of 41.4 ± 14.08 years. Reduced estimated glomerular filteration rate (eGFR; <60 ml/min/1.73 m²) was found in 24 patients (63%), while seven (18.4%) were diagnosed with nephrotic syndrome, another seven (18.4%) had sub-nephrotic range proteinuria, and four (10.5%) presented with microscopic hematuria. Two patients (5.2%) were already on maintenance hemodialysis when diagnosed with Sjögren's syndrome, and one patient was identified post-kidney transplant. Renal biopsies revealed various pathological findings, with chronic tubulointerstitial nephritis being the most common (N=4/11, 36.3%). Immunosuppressive therapy was given to 20 patients (56%), primarily steroids alone (N=8, 21%), while others received conservative management and supportive care.

Conclusion

Chronic tubulointerstitial nephritis is the most frequent form of renal involvement in Primary Sjögren's syndrome, with most cases diagnosed at advanced stages of kidney disease. This study underscores the importance of early and comprehensive renal evaluations in patients with Sjögren's syndrome, as early detection and timely treatment may help prevent progression to chronic kidney disease.

Learning points

Screening for renal involvement in primary Sjögren's syndrome (pSS) should include urinalysis, serum creatinine levels, and kidney biopsy when appropriate. A kidney biopsy showing characteristic findings, such as tubulointerstitial nephritis (TIN), should be considered an additional supportive criterion for the classification of pSS, as it can influence both treatment decisions and renal outcomes.

## Introduction

Sjögren's syndrome (SS) is a chronic autoimmune connective tissue disorder that primarily affects the lacrimal and salivary glands, with hallmark symptoms including keratoconjunctivitis sicca and xerostomia, collectively known as the sicca complex [1]. SS is categorized into primary and secondary forms. Secondary Sjögren's syndrome refers to cases where keratoconjunctivitis sicca, xerostomia, or both occur alongside another connective tissue disease or chronic inflammatory condition, such as rheumatoid arthritis, systemic lupus erythematosus, polymyositis, systemic sclerosis (scleroderma), or granulomatosis with polyangiitis. Sjögren-like symptoms may also appear in patients with infections like hepatitis C, human immunodeficiency virus (HIV), and human T-lymphotropic virus [2].

Primary Sjögren's syndrome (pSS) is the most common connective tissue disease after rheumatoid arthritis, affecting 0.3-3% of the population [3]. It is characterized by a predominant lymphocytic infiltrate around the epithelial cells of exocrine glands, as observed in salivary gland biopsies. Up to a quarter of patients may experience extraglandular manifestations, with the lungs, kidneys, nervous system, and skin being potentially affected [4]. Serologic findings in pSS typically include positive antinuclear antibodies (ANA), anti-SSA, and anti-SSB antibodies [5].

Renal involvement in pSS is often underdiagnosed, with reported prevalence rates ranging from 2% to 67% [6]. This wide range is likely due to differences in diagnostic criteria and the underdiagnosis of subclinical tubular disease, which cannot be detected using standard kidney function tests. Kidney disease in pSS poses a diagnostic challenge, as symptoms are often subtle and can appear before sicca symptoms. pSS affects the kidneys through lymphocytic infiltration of renal tubules or immune complex deposition, leading to a variety of clinical manifestations [7]. Depending on the anatomical and functional characteristics of the affected tubular epithelium, clinical presentations may range from asymptomatic urinary abnormalities, renal tubular acidosis (RTA), nephrogenic diabetes insipidus, and in rare cases, end-stage kidney disease due to chronic interstitial nephritis. The classic renal lesion in pSS is tubulointerstitial nephritis (TIN), with glomerular lesions such as membranoproliferative glomerulonephritis (MPGN) being less common and associated with cryoglobulinemia [8].

Tubulointerstitial nephritis is histologically characterized by a lymphocytic infiltrate around the tubules and fibrosis, and it rarely progresses to end-stage renal disease. Dysfunction of the H+/K+-ATPase pump, resulting in distal RTA and hypokalemia, is common in these patients (affecting nearly 50% in one study) [9]. The incomplete form of dRTA has a more subtle presentation and requires urinary acidification testing for diagnosis. In contrast, glomerulonephritis is caused by immune complex deposition in the glomeruli, often involving type II cryoglobulins, leading to membranoproliferative glomerulonephritis. This condition presents more abruptly than interstitial nephritis and is characterized by nephritic syndrome and impaired kidney function. Patients with pSS and glomerulonephritis have a poorer prognosis and may experience significant renal impairment if not treated promptly [10].

This study underscores the need for comprehensive renal evaluations in patients with Sjögren's syndrome to improve their health and quality of life. Early detection and appropriate treatment can help prevent the progression to chronic kidney disease.

## Materials and methods

Study design

This was a retrospective analysis conducted at two tertiary care private centers in North India - Max Super Speciality Hospital, Saket, New Delhi, India, and Fortis Gaur Hospital, Rajasthan, India. Data was collected from hospital information systems and nephrology outpatient medical records from 2013 to 2021. The search terms used to retrieve records included "Sjögren's syndrome", "Primary Sjögren's syndrome", "keratoconjunctivitis sicca", and "tubulointerstitial nephritis."

Inclusion criteria include patients diagnosed with primary Sjögren's syndrome based on the 2016 American College of Rheumatology/European League Against Rheumatism (ACR-EULAR) Classification Criteria, age over 18 years. Exclusions were applied to patients with a history of head and neck radiation therapy, active hepatitis C infection (with positive polymerase chain reaction), acquired immunodeficiency syndrome, sarcoidosis, amyloidosis, graft-versus-host disease, or IgG4-related disease.

Study procedure

Data collection included baseline demographics, clinical features, especially those related to renal involvement, laboratory investigations, and treatment details. Clinical parameters collected included gender, age, time from symptom onset to diagnosis, duration of pSS, renal involvement, initial symptoms, glandular and extraglandular symptoms, and treatment, including for nephrolithiasis. Laboratory parameters included in the study were urinalysis, complete blood count, biochemistry, arterial blood gas (ABG) analysis, abdominal ultrasound, and kidney or salivary gland histopathology, if available.

Biochemical tests that were included in the study were conducted using standard methods, and serum creatinine was measured by an isotope dilution mass spectrometry (IDMS)-traceable technique. Immunologic testing included anti-SSA and anti-SSB antibodies (detected using line immunoassay) and ANA (detected via immunofluorescence). Kidney biopsy reports were reviewed where available. 

Renal involvement was defined as one or more of the following [10]: complete distal RTA (cRTA), positive urine acidification test (UAT) without overt acidosis with or without hypokalemia (incomplete dRTA, iRTA), renal colic with findings of nephrolithiasis or nephrocalcinosis and overt dRTA or positive UAT, fanconi's syndrome not associated with any known cause, reduced creatinine clearance (<60 ml/min, CKD-EPI), proteinuria>500 mg/24 h, active urinary sediments and renal biopsy demonstrating histological features compatible with GN, interstitial nephritis, or both.

Data collection and statistical analysis

All data were collected, including demographics, clinical assessment, pathological findings, and serologic results. Continuous data was expressed as mean (SD) or median (range) values. 

Ethical approval 

The study protocol was approved by the institutional ethics committee of the two institutes where the study was conducted. The study adhered to the ethical standards of the Helsinki Declaration and complied with applicable Good Clinical Practice (GCP) guidelines.

## Results

Among the 38 patients, 18 (47.4%) had been diagnosed with pSS and were referred to a nephrologist for renal abnormalities at an average of 9.16 ± 8.29 months after their pSS diagnosis. The other 20 patients (52.6%) presented to a nephrologist with varying renal issues and were diagnosed with Sjögren's syndrome during the evaluation of renal dysfunction. The study population consisted of 36 female patients (94.7%) and two male patients (5.3%), with a mean age of 41.4 ± 14.08 years (range 18-72 years). Table 1 presents the baseline clinical characteristics, both renal and non-renal, as well as the laboratory parameters.

**Table 1:** Baseline characteristics of patients with primary Sjogren’s syndrome and renal involvement DM - diabetes mellitus; eGFR - estimated glomerular filtration rate; CKD - chronic kidney disease; dRTA- distal renal tubular acidosis; ANA - antinuclear antibody *Only a limited number of subjects received this test **Positive ANA defined as more than 1:80

Baseline characteristics and classification criteria	N (%)
Mean age (years)	41.4
Male	2 (5)
Female	36 (95)
DM	1 (2)
HTN	2 (5)
Overlap syndrome	6 (15.7)
Clinical features (non-renal)	19 (50)
Dry eyes only	14 (36.8)
Dry mouth only	3 (7.8)
Dry eyes + dry mouth	14 (36)
Cutaneous Vasculitis	4 (10.5)
Arthralgia	17 (44)
Positive Schirmer’s test or Rose Bengal score *	18/18
Positive salivary gland biopsy* (n=1)	1 (100)
Palpable glands	5 (13.1)
Dental involvement (dental caries/plaque/gingivitis)	6 (15.7)
Hypokalemia	15 (39.4)
Hypokalemic paralysis	10 (26.3)
Proteinuria	10 (26.3)
Hematuria	4 (10.5)
Mean serum creatinine at presentation (mg/dl) (for non-dialysis dependent patients)	1.51
Nephrolithiasis	8 (21)
Nephrotic syndrome	7 (18.4)
Subnephrotic proteinuria	6 (15.7)
Microscopic hematuria	4 (10.5)
Reduced eGFR (<60 ml/min/1.73 m^2^)	24 (63.1)
CKD 3-5	21 (55.2%)
CKD 5 on maintenance hemodialysis	2 (5.2%)
Post renal transplant	1 (2%)
Nephrolithiasis	8 (21)
Distal RTA	11 (28.9)
Dialysis dependency	2 (5)
ANA positivity**	24 (63.8)
Anti-SSA+	33 (86)
Anti-SSB+	13 (34)
Low C3* (n=26)	2 (7.7)
Raised ESR	11/38
Rheumatoid factor positivity* (n=20)	2 (10)

Nineteen patients had constitutional symptoms (fever, night sweats, weight loss), while 31 (81.5%) exhibited sicca symptoms. Among those with sicca, 14 (36.8%) had dry eyes only, three (7.8%) had only dry mouth, and 14 (36.8%) had both dry eyes and dry mouth. Extraglandular involvement, aside from the kidneys, included cutaneous manifestations (N=4, 10.5%), interstitial lung disease (N=1, 2.6%), seronegative arthritis (N=2, 5.2%), and dental issues (N=6, 15.7%).

Additionally, three patients (7.8%) tested negative for both anti-SSA and anti-SSB antibodies. ANA positivity was observed in 24 of the 38 patients (63.8%), and two patients (5.2%) were positive for rheumatoid factor. Five patients (13.1 %) had palpable salivary glands, and one (2.6%) of them had a biopsy-confirmed diagnosis of focal lymphocytic sialadenitis.

Twenty-four patients (63.1%) presented with an incidental finding of elevated serum creatinine, seven (18.4%) with nephrotic syndrome, seven (18.4%) had sub-nephrotic range proteinuria, and four (10.5%) had microscopic hematuria. Hypokalemia was observed in 15 patients (39.4%), 11 (28.9%) of whom were diagnosed with distal renal tubular acidosis, and 10 (26.3%) had a history of hypokalemic paralysis. Nephrolithiasis was present in eight patients (21%).

Mean serum creatinine at the time of presentation for non-dialysis dependent patients were 1.51 ±0.64 mg/dl. Additionally, two patients (5.2%) with CKD stage 5 were on maintenance hemodialysis and were later diagnosed with Sjögren's syndrome. One patient (2.6%), who presented with post-renal transplant graft dysfunction, was diagnosed with pSS during the evaluation.

Kidney biopsy

Biopsy specimens were available for 11 of the 38 patients (28.9%) (Table 2). Among these, four patients had chronic glomerulosclerosis, and another one (N=1/11, 9.09%) had hypertensive nephrosclerosis with thrombotic microangiopathy (TMA) and no significant interstitial involvement. Two patients (N=2/11, 18.1 %) were diagnosed with mesangioproliferative glomerulonephritis, both associated with focal segmental glomerulosclerosis (FSGS) and negative immunofluorescence. One patient (N=1/11, 9.09%) had proliferative glomerulonephritis along with acute interstitial nephritis, and another one (N=1/11, 9.09%) had focal segmental glomerulosclerosis. One patient's biopsy showed normal renal findings (N=1/11, 9.09%).

**Table 2:** Histological manifestations of renal involvement in primary Sjögren's syndrome FSGS - focal segmental glomerulosclerosis; TMA - thrombotic microangiopathy *%, out of the 11 patients whose biopsy was done

S. No.	Pathology type	No of cases (N, %)*
1	Chronic interstitial nephritis	4 (36.3%)
2	Chronic glomerulosclerosis	1 (9.09%)
3	Hypertensive nephrosclerosis with TMA	1 (9.09%)
4	Mesangioproliferative glomerulonephritis	2 (18.1%)
5	Proliferative GN with acute interstitial nephritis	1 (9.09%)
6	FSGS	1 (9.09%)
7	NORMAL	1 (9.09%)

Treatment outcomes

The treatment and follow-up of the patients were also analysed and summarised in Table 3. Out of the 38 patients, 17 (44.7%) received only symptomatic treatment. Eight patients (21%) were started on oral steroids. One patient (2.63%) with mesangioproliferative glomerulonephritis was treated with a combination of rituximab and steroids. Additionally, three patients (7.8%) were started on azathioprine, seven (18.4%) were treated with mycophenolate mofetil (MMF), and one patient (2.63%) received MMF followed by azathioprine.

**Table 3:** Treatment options for patients with primary Sjögren's syndrome and renal involvement

S.No.	Treatment received	N (%)
1.	Symptomatic treatment	17(44.7)
2.	Oral steroids only	8 (21%)
3.	Rituximab and steroids	1 (2.6%)
4.	Azathioprine	7 (18.4%)
5.	Mycophenolate mofetil	7 (18.4%)
6.	Mycophenolate mofetil followed by azathioprine	1 (2.6%)

Out of 38 patients, follow-up data of 18 patients were available. Of the 18 patients, the mean creatinine at presentation was 1.92 ± 0.73 mg/dl and at three months was 2.2 ± 1.09 mg/dl. Over the course of follow-up, five patients (N=5/18, 27.7%) developed renal failure requiring dialysis, out of which one had renal transplantation done and had a stable graft function. Three CKD patients died during follow-up, and seven patients were lost to follow-up after one year.

One patient presented to us with post-renal transplant status and had stable graft function but developed RTA after one year of renal transplant, for which they received supportive treatment involving correction of acidosis and hyperkalemia with bicarbonate and potassium supplements.

There were seven patients who presented with nephrotic syndrome with an average spot urine protein creatinine ratio of 3±1.4 g/day. They all received prednisolone and HCQ, and one patient also received tacrolimus followed by azathioprine. The mean UPCR after three months of treatment was 0.86±0.12g/day.

## Discussion

This study reaffirms previous findings that patients with primary Sjögren's syndrome (pSS) can experience significant renal involvement. The majority of patients in our series were middle-aged females. Among these, 11 patients (28.9%) had elevated inflammatory markers (as indicated by ESR), but only two out of 20 (10%) tested were positive for rheumatoid factor. Thirty-six patients (95%) tested positive for anti-SSA and/or SSB antibodies, and 24 patients (63.8%) were positive for ANA.

A cross-sectional study by Luo et al. [11] found that elevated ESR was significantly more common in pSS patients with renal involvement compared to those without renal involvement. However, there were no significant differences in autoantibody profiles between the two groups, including ANA positivity (ANA >1:320), anti-SSA/Ro60 positivity, and anti-La/SSB positivity. Notably, anti-SSA/Ro52 positivity was much higher in patients without renal involvement. This contrasts with another study by Luo et al [5], which reported that pSS patients with renal involvement had significantly higher levels of rheumatoid factor (RF) compared to those without renal involvement, though ESR levels did not differ significantly between the two groups.

pSS can present with a variety of renal manifestations. Our study reinforces existing evidence that pSS can lead to diverse renal issues, including less common glomerular involvement. In our study, the most frequent renal issue was decreased eGFR (N=24, 63.1%), and 18.4% (N=7) of patients presented with nephrotic syndrome. A cross-sectional study evaluating 60 pSS patients found that 27% had significant laboratory abnormalities indicating tubular or glomerular pathology [12]. Additionally, a retrospective study following 471 patients over a decade identified 4.2% with overt renal disease, with 18 of these patients undergoing renal biopsy [13].

In the present study, 18 out of 38 patients (47.3%) diagnosed with primary pSS were found to have renal involvement upon screening, with a mean duration of 9.16 ± 8.29 months. The remaining patients were diagnosed with pSS through evaluation of renal dysfunction. Tubulointerstitial inflammation in pSS is known to lead to interstitial fibrosis and CKD [14]. Although rare, glomerulonephritis associated with pSS can occur and often develops later in the disease course. The low prevalence may be attributed to the infrequent use of routine renal biopsies. The proposed mechanism involves the deposition of immune complexes composed of cryo-precipitating monoclonal immunoglobulin M (IgM) along with polyclonal IgG and IgA. However, glomerular disease may also occur without circulating cryoglobulins, presenting as membranous or membranoproliferative lesions [15]. In some cases, renal manifestations can be the initial clinical presentation, while pSS may also be diagnosed much later, such as after renal transplantation or the development of ESRD.

A renal biopsy was conducted in 11 out of 38 patients (28.9%), with the most frequent histopathological finding being chronic interstitial nephritis, a result consistent with other studies [4,16]. Kidder et al. reported a series of 25 patients where Sjögren's syndrome preceded renal manifestations by a median of 5.5 years. In this series, 22 patients were female, with a median age of 55 years at the time of biopsy. Thirteen patients exhibited proteinuria greater than 1 g, and 21 patients had an eGFR below 60 mL/min/1.73 m², with over half of these patients having an eGFR below 30 mL/min/1.73 m².

Renal involvement in pSS commonly presents as TIN, characterized by significant lymphocytic infiltration, primarily of CD4+ T cells, as a result of epithelial disease [16]. In our study, we reported one case each of chronic glomerulosclerosis, hypertensive nephrosclerosis with TMA, mesangio-proliferative glomerulonephritis with FSGS, proliferative glomerulonephritis with acute interstitial nephritis, and FSGS. Another series observed renal involvement in 4.9% of 715 Sjögren's syndrome patients, with glomerular involvement being more common than tubulointerstitial disease. In that series, 37% had interstitial nephritis alone, 49% had glomerulonephritis alone, and 14% had both [10]. Prognosis tends to be worse in patients with predominantly glomerular involvement, who have lower survival rates and a higher incidence of lymphoma compared to those with tubulointerstitial involvement [4].

The optimal therapy for pSS remains uncertain, as no systemic immunosuppressive treatment has been definitively proven effective. Treatment strategies are largely extrapolated from therapies used in other inflammatory conditions, such as SLE, and small open-label studies [7]. Systemic steroids are often initiated empirically. A recent review by Aiyegbusi et al. on managing renal involvement in pSS suggests that treatment is largely dependent on the specific renal disease process [7]. Immunosuppressive therapies aimed at reducing inflammation and preventing fibrosis may be considered in certain cases.

Treatment options are disease-specific and may include corticosteroids, antiproliferative agents, calcineurin inhibitors, cyclophosphamide, and B-cell depletion therapy. For patients who are intolerant to steroids or have refractory disease, alternative treatments may be considered. Mycophenolate mofetil (MMF) inhibits the proliferation of B and T lymphocytes, which are key contributors to the lymphocyte-rich infiltration seen in pSS. In a case series of pSS patients with TIN, MMF was shown to significantly improve kidney function [17].

Although our sample size was small, the patients in our study who were diagnosed early, before the progression to ESRD, showed responsiveness to treatment and either maintained or improved renal function during follow-up. This underscores the importance of early detection and timely treatment.

This study has a number of limitations. Firstly, the number of cases is small and spread over almost a decade, reflecting the rare nature of pSS and renal involvement. Secondly, due to the lack of a definitive treatment protocol, there may be differences in outcome results as per individual physicians' management practices.

## Conclusions

Considering the impact of progressive renal disease in pSS patients, particularly those who develop severe renal impairment before diagnosis, there should be a stronger focus on screening for renal involvement, with a low threshold for early nephrology referral and kidney biopsy. Unfortunately, many cases go undetected until significant renal dysfunction has already developed, highlighting the critical need for early detection and prompt, aggressive treatment. There is also a need for comprehensive data to establish clear guidelines for the treatment of these patients.
